# Comprehensive Transcriptomic and Bioinformatic Analysis of the Mechanism of Buzhong Yiqi Decoction in the Improvement of Diabetic Nephropathy

**DOI:** 10.2174/0118715303379062250327184950

**Published:** 2025-04-21

**Authors:** Xixu Zhang, Wei Wei, Ziyu Liu, Hao Gao, Fengyi Guo, Donglin Liu, Yuanping Yin, Xiao Yang

**Affiliations:** 1 The Second Affiliated Hospital of Liaoning University of Chinese Medicine, Shenyang, Liaoning 110034, China;; 2 Liaoning University of Chinese Medicine, Shenyang, Liaoning 110847, China

**Keywords:** Diabetic nephropathy, DN rat model, buzhong Yiqi decoction, transcriptomics, bioinformatic analysis, Th1/Th2 immune cell

## Abstract

**Background:**

Buzhong Yiqi decoction (BZYQ) is a classical traditional Chinese formula that has shown potential in alleviating diabetic nephropathy (DN). However, its underlying mechanisms remain unclear.

**Objective:**

We aimed to explore the potential targets and mechanisms of action of BZYQ in DN.

**Materials and Methods:**

A DN rat model was induced using a high-fat and high-sugar diet combined with intraperitoneal injection of streptozotocin (STZ), followed by treatment with different doses of BZYQ. Initially, the protective effects of BZYQ on renal tissue were assessed by measuring fasting blood glucose (FBG), total cholesterol (TC), triglycerides (TG), 24-hour urinary total protein (24h-UTP), urinary albumin (ALB), and serum creatinine (SCr) after administration, along with performing hematoxylin-eosin (HE) staining. Subsequently, transcriptomics and bioinformatics approaches were employed to identify the action targets and potential mechanisms of BZYQ in DN rats. Finally, real-time PCR and Western blot analyses were conducted to validate key targets and mechanisms.

**Results:**

We observed significant improvements in renal injury in DN rats treated with medium and high doses of BZYQ. Transcriptomic and bioinformatic analyses identified NLRP3, ASC, caspase-1, GSDMD, IL-1β, and IL-18 as hub genes, along with differential expression of immune-related transcription factors T-bet and GATA-3 in various transcriptomes. In the validation phase, the mRNA and protein expressions of NLRP3, ASC, caspase-1, GSDMD, IL-18, IL-1β, and T-bet were significantly elevated in the DN rat model group, while GATA-3 mRNA and protein levels were significantly decreased; BZYQ was able to reverse these changes.

**Conclusion:**

BZYQ has been found to have a protective effect on renal tissue damage in DN rats, potentially related to the inhibition of NLRP3 inflammasome pathway activation and the improvement of Th1/Th2 immune cell balance.

## 1 INTRODUCTION

Diabetic nephropathy (DN) is a clinical syndrome characterized by persistent proteinuria and progressive decline in kidney function, commonly seen in the mid to late stages of diabetes. Approximately 20%-50% of diabetic patients develop DN, making it a primary cause of end-stage renal disease (ESRD) among diabetes sufferers [1]. According to epidemiological studies, it is estimated that by 2030, the number of DN patients worldwide will exceed 100 million, and by 2045, it could reach 700 million, with around 30%-50% of diabetic patients in China potentially facing the risk of developing DN [2, 3]. Therefore, delaying the progression of DN is of significant importance for improving the quality of life of diabetic patients.

Traditional Chinese medicine (TCM), as a safe, effective, and alternative therapy, represents a promising approach to treating DN [4]. Buzhong Yiqi decoction (BZYQ) is a traditional Chinese formula composed of eight herbs: Huangqi (the dried roots of *Astragalus membranaceus* (Fisch) Bge), Renshen (the dried roots of *Panax ginseng* C.A. Meyer (Araliaceae)), Baizhu (the dried rhizome of *Atractylodes macrocephala* Koidz (Asteraceae)), Chenpi (the dried mature fruit peels of *Citrus reticulata* and *Citrus sinensis*), Shengma (the dried rhizome of *Cimicifuga foetida* L.), Chaihu (the dried roots of *Bupleurum falcatum* L.), Danggui (the dried roots of *A. sinensis* (Oliv.) Diels), and Zhigancao (the processed dried roots or rhizomes of *Glycyrrhiza uralensis* Fisch., G. *inflata* Bat., or *G. glabra* L.). It is known for its effects related to tonifying Qi, strengthening the spleen, raising Yang, and uplifting prolapsed organs. In recent years, several studies have reported that BZYQ can reduce urinary protein and blood glucose levels, improve kidney function, and achieve promising clinical outcomes in delaying the progression of DN [5-7]. However, the underlying mechanisms remain unclear and require further investigation.

With the advancement of high-throughput sequencing technology, transcriptomics can help us precisely understand gene expression patterns and regulatory mechanisms [8]. Due to the complex multi-component and multi-target regulatory mechanisms of TCM in treating diseases, transcriptomics has been widely applied in pharmacological mechanism research in recent years. By performing high-throughput transcriptomic sequencing, transcriptomic expression profiles before and after treatment with TCM can be obtained [9]. Subsequently, through bioinformatics analysis techniques, we can identify differentially expressed genes (DEGs), construct gene co-expression networks, and conduct Gene Ontology (GO) enrichment analyses, thereby comprehensively revealing how TCM affects the gene expression patterns of specific diseases and enhancing our understanding of their complex therapeutic mechanisms [10, 11].

Therefore, we established a DN rat model to observe the comprehensive effects of BZYQ intervention on the disease. We then utilized transcriptomic sequencing technology to identify DEGs in the two datasets before and after treatment, further employing bioinformatics techniques to screen for key targets and mechanisms. Finally, we conducted experimental validation of the key targets to elucidate the effects of BZYQ on the progression of DN and its potential mechanisms.

## 2 MATERIALS AND METHODS

### 2.1 Chemicals and Reagents

Buzhong Yiqi decoction (BZYQ) was purchased from the Second Affiliated Hospital of Liaoning University of TCM, containing eight traditional Chinese herbs, including 18g Huangqi, 9g Baizhu, 9g Renshen, 6g Chenpi, 6g Shengma, 6g Chaihu, 6g Zhigancao, and 6g Danggui. The positive control drug was Losartan potassium, a chemical drug with the national drug approval number J20180054. Serum creatinine (SCr) and other reagent kits were provided by Jiangsu Meimian Industrial Co., Ltd. and Nanjing Jiancheng Bioengineering Institute. Antibodies for NLRP3, ASC, caspase-1, GSDMD, and IL-1β were supplied by Beijing Bioss. The instruments used included the M200 PRO microplate reader from Switzerland (Tecan), the QuantStudio 3 PCR amplifier from the USA (ABI), and the Tanon 5200 chemiluminescence detection system from Shanghai Tanon Science & Technology Co., Ltd.

### 2.2 Animal Culture and Grouping

One hundred male Sprague-Dawley (SD) rats (200 ± 20g, 8 weeks old) were housed in an environment with a temperature of 25°C and a relative humidity of 55-60%, under a 12-hour light/dark cycle. After one week of acclimatization, the rats were randomly divided into a control group (NC, 10 rats) and a model group (MC, 90 rats). The control group was fed a regular diet, while the remaining groups received a high-fat and high-sugar diet (66.5% standard rodent chow + 10% lard + 20% sucrose + 2.5% cholesterol + 1% sodium cholate). After six weeks, all rats were fasted for 12 hours, but were allowed water. The model group received a single intraperitoneal injection of a 1% STZ solution prepared in citric acid-citrate buffer (pH=4.2-4.5) at a dose of 30 mg/kg, while the control group received an equal volume of the buffer. After 72 hours, blood was collected from the tail vein to measure blood glucose. If fasting and/or random blood glucose levels reached ≥ 16.7 mmol/L on three separate occasions, and 24-hour urinary protein (UTP) exceeded 30 mg in urine collected from metabolic cages, the rat DN model was considered successfully established [12]. Rats successfully induced were randomly assigned to the model group, low-dose BZYQ group (BZYQ-L), medium-dose BZYQ group (BZYQ-M), high-dose BZYQ group (BZYQ-H), and Losartan potassium group (LP), totaling five groups with 18 rats in each group. The treatment groups received respective doses of Losartan potassium (4.5 mg/kg/d) and BZYQ (L-2.97, M-5.94, H-11.88g/kg/d) *via* oral gavage at a volume of 10 mL/kg. The blank control and model groups were given an equal volume of distilled water once daily. After 10 weeks, the rats were euthanized using sodium pentobarbital. This study adhered to the ethical guidelines set by the ethics committee of Liaoning University of TCM (ethics approval number: LZYY240902). All animal housing and experiments were conducted in strict accordance with the institutional guidelines for the care and use of laboratory animals. Experimental animals were provided by Liaoning Changsheng Biotechnology Co., Ltd., with animal license number SCXK (Liao) 2020-0001.

### 2.3 Blood Glucose and Lipid Tests

Blood was drawn from the tail vein of the rats, allowed to sit, and then centrifuged at 3000 rpm for 10 minutes to obtain serum. An automatic biochemical analyzer was used to measure fasting blood glucose (FBG), total cholesterol (TC), and triglycerides (TG).

### 2.4 Detection of Renal Function

Urine samples from the rats were collected using metabolic cages. After centrifugation, 24-hour urinary microalbumin (24h-UAlb) and urinary albumin levels were measured. Blood was collected from the abdominal aorta and centrifuged at 3000 rpm for 15 minutes to isolate serum, which was then analyzed for serum creatinine (SCr) levels according to the kit instructions. The remaining serum samples were stored at -80°C for future testing.

### 2.5 Renal HE Staining

The left kidneys from each group of rats were aseptically removed and fixed in 4% paraformaldehyde for 24 hours. After washing with phosphate buffer solution (PBS) and dehydration, the tissues were routinely embedded in paraffin, and continuous sections (4 μm) were prepared. The sections were stained with hematoxylin and eosin (HE), and after sealing, the pathological changes in renal tissues were observed under a light microscope.

### 2.6 RNA Sequencing Analysis

Total RNA from the kidney tissues of the control group, model group, and high-dose BZYQ group was extracted using TRIzol. Before further analysis, the purity, concentration, and integrity of the total RNA samples were assessed. Next, library preparations were sequenced using the Illumina HiSeqTM 4000 platform by Sangon Biotech (Shanghai, China), and raw read counts were generated. FastQC was used to assess the quality of the sequencing data, and quality trimming was performed using Trimmomatic to obtain relatively accurate and clean data. The clean data were aligned to the rat reference genome (Rnor 6.0) using HISAT2 software, and mapping information was generated. Guided by the reference genome, transcriptome assembly was performed, and gene expression levels were evaluated using StringTie and known gene models. DEGs were identified using DESeq2, with the criteria set as q-value < 0.05 and |FoldChange| > 2 [13].

### 2.7 Bioinformatic Analysis

DEGs from the MC *vs*. NC group and the BZYQ-H *vs*. MC group were input separately into the Venny2.1.0 software, and the intersection of the mapped results represented the potential targets of BZYQ in acting on DN. Protein-protein interaction (PPI) network analysis was conducted using the STRING database by setting the species to *Rattus norvegicus* and the minimum required interaction score to 0.40. The results were further visualized using Cytoscape 3.10.1 software. The MCODE plugin was used for topological analysis of the network, and the key components identified were considered hub genes of BZYQ's effect on DN. Finally, Gene Ontology (GO) and Kyoto Encyclopedia of Genes and Genomes (KEGG) enrichment analyses were performed using the David website (https://david.ncifcrf.gov/), and the results were visualized through the Bioinformatics website (https://www.bioinformatics.com.cn/) [14].

### 2.8 Real-time PCR (qPCR)

Total RNA was extracted from kidney tissues using TRIzol reagent, following the manufacturer's instructions. The absorbance at 260 nm and 280 nm was measured using a UV spectrophotometer, and the purity and concentration of the RNA were calculated. Genomic DNA was removed using a reverse transcription kit, and the reaction system was prepared on ice. After incubation at 42°C for 2 minutes, the reaction mixture was added and incubated for reverse transcription at 42°C for 15 minutes, followed by incubation at 85°C for 5 minutes to synthesize cDNA. Glyceraldehyde-3-phosphate dehydrogenase (GAPDH) gene's mRNA levels were used as an internal control. The PCR amplification was performed using SYBR Green with the following conditions: pre-denaturation at 95°C for 30 seconds, denaturation at 95°C for 5 seconds, and annealing at 60°C for 30 seconds, repeated for 40 cycles, followed by denaturation at 95°C for 15 seconds, cooling at 60°C for 1 minute, another denaturation at 95°C for 15 seconds, and a final hold at 50°C for 30 seconds. Quantitative analysis was performed using the 2-ΔΔCt method, and primer sequences are listed in Supplementary Table **1**.

### 2.9 Western Blotting (WB) Analysis

The protein concentration was quantified using a BCA protein assay kit. After determining the protein concentration of thyroid tissue using the BCA kit, proteins were denatured with 5× loading buffer, with about 10 μg of protein loaded per well. Electrophoresis was performed at 160 V for 30 minutes, followed by wet transfer at 100 V constant voltage for 30 minutes. After transfer, the membrane was washed 3 times with TBST, with each wash lasting 10 minutes, and then blocked with 5% skim milk for 1 hour. After blocking, the membrane was washed 4 times with TBST, with each wash lasting 10 minutes, followed by the addition of the primary antibody and overnight incubation at 4°C. The membrane was then washed 3 times with TBST, with each wash lasting 15 minutes, followed by the addition of the HRP-conjugated secondary antibody and shaking at room temperature for 1 hour. The membrane was again washed 3 times with TBST, with each wash lasting 15 minutes, and then developed with ECL working solution, exposed, and the bands were saved for grayscale analysis.

### 2.10 Statistical Analysis

Data were processed and analyzed using GraphPad Prism 9.0 software. For data meeting normality and homogeneity of variance, results were expressed as mean ± standard deviation (±s), and comparisons between groups were made using one-way ANOVA. Normality was assessed using the Shapiro-Wilk test, and variance homogeneity was evaluated using Levene’s test. If normality and homogeneity of variance were not met, a non-parametric rank-sum test was used. A *P*-value < 0.05 was considered statistically significant.

## 3 RESULTS

### 3.1 BZYQ Reduced Blood Glucose Levels and Lipids in DN Rats

Compared to the control group, the model group showed significant increases in fasting blood glucose (FBG), total cholesterol (TC), and triglycerides (TG) (*P* < 0.05). In contrast, FBG, TC, and TG were decreased in the BZYQ-M, BZYQ-H, and LP groups compared to the model group (*P* < 0.05). The results are shown in Figs. (**1**, **1b**-**d**).

### 3.2 BZYQ Ameliorated Renal Function in DN Rats

**Fig. (1):**
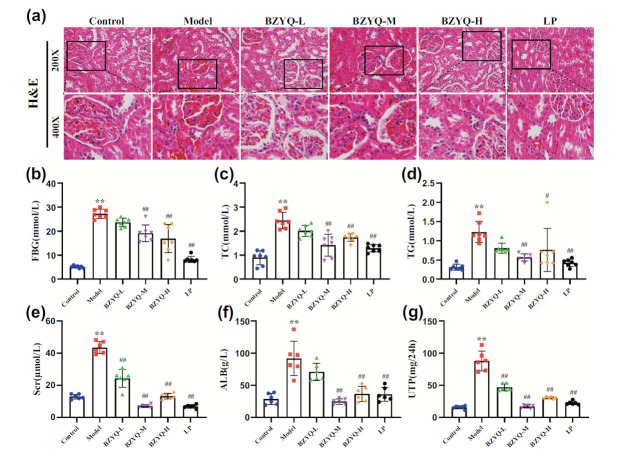
The effects of BZYQ on DN rats. Histopathological examination of H&E staining kidney tissues (**a**), fasting blood glucose (**b**), total cholesterol (**c**), triglycerides (**d**), serum creatinine (**e**), urinary albumin (**f**), 24-hour urinary microalbumin (**g**). *P < 0.05 *versus* the Control group. **P < 0.01 *versus* the Control group. ^#^*P* < 0.05 *versus* the Model group. ^##^*P* < 0.01 *versus* the Model group.

Compared to the control group, the model group exhibited significant increases in SCr, Alb, and 24h-UAlb (*P* < 0.05). In contrast, the BZYQ-M, BZYQ-H, and LP groups demonstrated significant reductions in SCr and Alb compared to the model group (*P* < 0.05). Additionally, the 24h-UAlb levels in all BZYQ groups and the LP group were significantly reduced (Fig. **1e**-**g**).

### 3.3 BZYQ Reduced Pathological Injury in the Kidneys of DN Rats

In the control group, the kidney structure was normal, with no inflammatory cell infiltration, and normal glomeruli, renal tubules, and interstitial tissues were observed. In the model group, the glomerular capillary loops were enlarged, the basement membrane was thickened, the renal tubular epithelial cells were shed, there was significant infiltration of inflammatory cells, and collagen fibers were deposited in the renal interstitium. In the BZYQ groups, pathological kidney damage was significantly improved (Fig. **1a**).

### 3.4 Transcriptomic and Bioinformatic Analysis of BZYQ in DN Rats

**Fig. (2):**
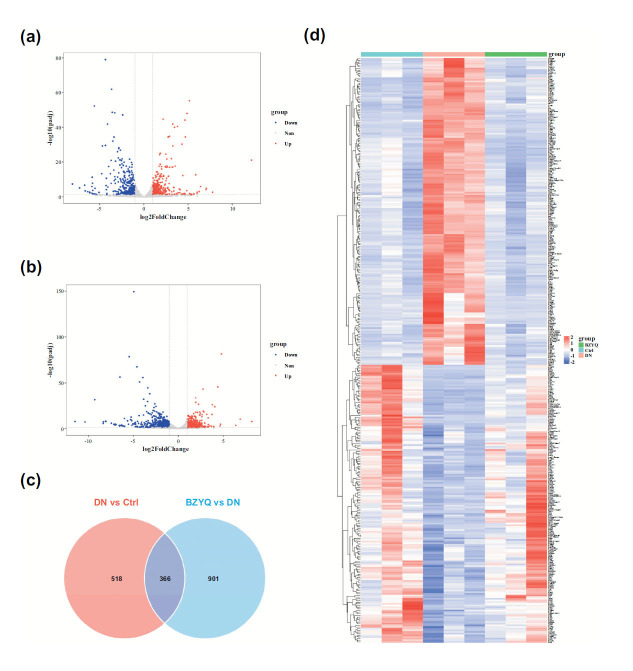
Transcriptomic analysis of BZYQ against DN. Volcanic map of differentially expressed genes between the model and control groups (**a**), volcanic map of differentially expressed genes between the BZYQ-H and model groups (**b**), Venn diagram of BZYQ in regulating DN (**c**), heatmaps of differentially expressed genes based on the RNA-seq dataset (**d**).

Using RNA-Seq analysis on the kidney tissues of rats in the control group, model group, and BZYQ-H group, we explored the protective genes and pathways involved in the anti-DN effects of BZYQ. Sequencing was performed on mRNA from three replicates of kidney samples from the control, model, and BZYQ-H groups. A total of 15,316 genes were identified (accounting for 46.58% of the 32,883 genes in the Rnor_6.0 genome), and these genes were expressed in at least one sample. To identify DEGs, we used a cutoff of q-value < 0.05 and a |FoldChange| > 2 for gene expression in the control, model, and BZYQ-H groups. Pairwise comparisons were conducted between the model and control groups, as well as between the BZYQ-H and model groups. Results showed 844 DEGs in the kidney tissues of DN model rats, and 1,267 DEGs were identified after BZYQ treatment. Overall, 404 upregulated and 480 downregulated DEGs were identified between the model and control groups (Fig. **2a**), and 664 upregulated and 603 downregulated DEGs were identified between the BZYQ-H and model groups (Fig. **2b**). Next, we subjected the DEGs from both datasets to a Venn diagram analysis, which identified 366 intersecting targets (Fig. **2c**). Further screening of the DEGs revealed genes with opposite expression patterns in both datasets. In summary, after DN modeling, 358 related DEGs were reversed by BZYQ-H treatment (Fig. **2d**). These genes were considered potential targets for BZYQ’s anti-DN effects and were subjected to further bioinformatic analysis.

**Fig. (3):**
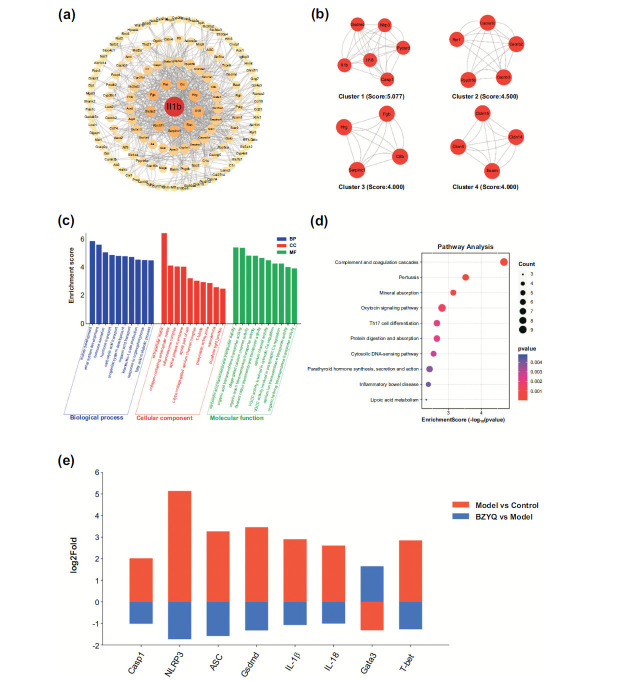
Bioinformatic analysis of BZYQ against DN. PPI network. The intensity of the color represents the degree of significance (**a**), the top four clusters of MCODE analysis score (**b**), the top 10 significantly enriched terms of GO analysis (**c**), the top 10 significantly enriched KEGG pathways (**d**), the expression of hub genes based on RNA-seq datasets (**e**).

Using STRING and Cytoscape software, we constructed a PPI network with these potential targets, which included 239 nodes and 600 edges (Figs. **3** and **3a**). Enrichment analysis indicated that the mechanism by which BZYQ treats DN mainly involves extracellular matrix, kidney development, carboxylic acid transmembrane transporter activity, renal system development, and organic acid transmembrane transporter activity (Fig. **3c**). These pathways are predominantly centered on the regulation of complement and coagulation cascades, Th17 cell differentiation, oxytocin signaling pathway, and cytosolic DNA-sensing pathway (Fig. **3d**). MCODE analysis identified four core subnetworks, with higher scores indicating greater importance of the respective clusters. The hub genes in the highest-scoring subnetwork, which we considered essential therapeutic targets for BZYQ in treating DN, included NLRP3, ASC, caspase-1, GSDMD, IL-1β, and IL-18 (Fig. **3b**). Interestingly, we also identified two transcription factors, T-bet and GATA-3, which play crucial roles in Th cell differentiation and function, exhibiting varying degrees of opposite expression changes after DN modeling and BZYQ intervention (Fig. **3d**).

### 3.5 BZYQ Restrained the Activation of the NLRP3 Inflammasome/Pyroptosis Pathway

**Fig. (4):**
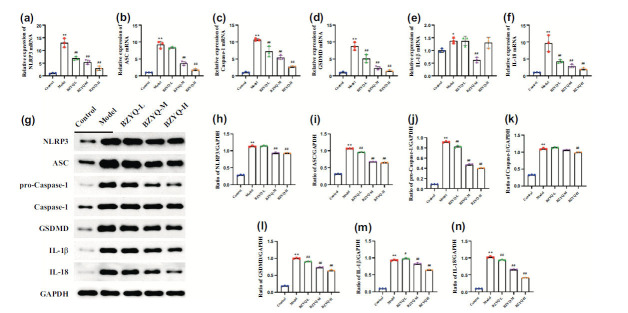
The effects of BZYQ on the NLRP3 inflammasome/pyroptosis pathway. Relative mRNA expression of NLRP3, ASC, Caspase-1, GSDMD, IL-1β, and IL-18 in the kidney as measured by qPCR (**a-f**), the protein expressions of NLRP3, ASC, pro-Caspase-1, Caspase-1, GSDMD, IL-18, and IL-1β in the kidney as identified through Western blotting with GAPDH as the internal reference (**g-n**). *P < 0.05 *versus* the Control group. **P < 0.01 *versus* the Control group. ^#^*P* < 0.05 *versus* the Model group. ^##^*P* < 0.01 *versus* the Model group.

Compared to the control group, the mRNA and protein levels of IL-18, IL-1β, NLRP3, ASC, pro-caspase-1, caspase-1, and GSDMD in the kidney tissues of model group rats were significantly elevated (*P* < 0.05). In comparison to the model group, the BZYQ groups reversed this trend (Fig. **4**).

### 3.6 BZYQ Regulated the Th1/Th2 Immunological Balance-related Factors

**Fig. (5):**
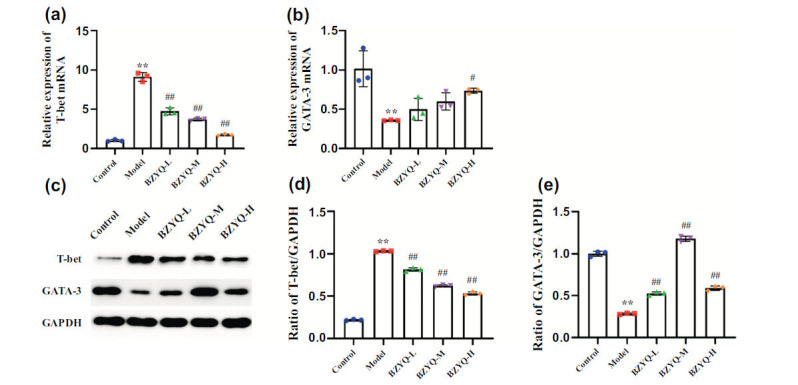
The effects of BZYQ on the Th1/Th2 immunological balance-related factors. Relative mRNA expression of T-bet and GATA-3 in the kidney as measured by qPCR (**a-b**), the protein expressions of T-bet and GATA-3 in the kidney as identified through Western blotting with GAPDH as the internal reference (**c-e**). *P < 0.05 versus the Control group. **P < 0.01 *versus* the Control group. ^#^P < 0.05 *versus* the Model group. ^##^*P* < 0.01 *versus* the Model group.

Compared to the control group, the mRNA and protein levels of T-bet in the kidney tissues of the model group rats were significantly increased (*P* < 0.05), while GATA-3 mRNA and protein levels were significantly decreased (*P* < 0.05). Compared to the model group, the BZYQ groups showed a decrease in T-bet mRNA and protein levels, along with an increase in GATA-3 mRNA and protein levels (*P* < 0.05) (Fig. **5**).

## 4 DISCUSSION

Diabetic nephropathy (DN) is a severe microvascular complication characterized by key pathological changes, such as thickening of the glomerular basement membrane, mesangial matrix accumulation, glomerulosclerosis, tubulointerstitial inflammation, and fibrosis, which eventually lead to renal failure [15]. Buzhong Yiqi decoction (BZYQ) is a traditional Chinese prescription, and recent studies have shown that its various active ingredients can improve DN. This study used a DN rat model to observe the protective effects of BZYQ on kidney injury. The results demonstrated that after the establishment of the DN model, indicators of kidney function, including serum creatinine (SCr), albumin (Alb), and 24-hour urinary albumin (24h-UAlb), were significantly elevated, and there were increased fasting blood glucose (FBG), total cholesterol (TC), and triglycerides (TG) levels. Pathological damage to the kidney tissue was also evident, confirming the successful construction of the diabetic nephropathy rat model. Compared to the model group, BZYQ significantly reduced urinary protein, serum creatinine, blood glucose, and lipid levels, and improved renal injury. Additionally, we found that the effect of BZYQ on DN improvement was dose-dependent.

The pathogenesis of DN is highly complex, and it is currently believed to result from the combined effects of multiple factors on genetic background, including oxidative stress, inflammatory responses, metabolic disorders, and hemodynamic abnormalities [16-18]. However, with further research, increasing evidence indicates that inflammatory responses play a crucial role in the development and progression of DN. Inhibiting inflammatory mediators, signaling pathways, and downstream products involved in the inflammatory process can significantly improve renal injury in DN [19]. Previous studies by our research group have shown that BZYQ could alleviate immune dysregulation in autoimmune thyroiditis mice by reducing inflammatory responses [20, 21]. Other studies have confirmed that BZYQ can treat diseases, such as allergic rhinitis, osteoporotic fractures, and obesity, by alleviating inflammation [22-24]. However, there are few reports on whether BZYQ can treat DN by modulating inflammatory responses, and the mechanism remains to be clarified.

Transcriptomics can comprehensively reveal the changes in gene expression profiles before and after treatment. Combined with bioinformatic analysis, it helps accurately identify drug target genes and mechanisms of action. The enrichment analysis results showed BZYQ to regulate multiple activities and pathways involved in DN, including extracellular matrix, kidney development, carboxylic acid transmembrane transporter activity, renal system development, Th17 cell differentiation, oxytocin signaling pathway, and cytosolic DNA-sensing pathway. The identification of hub gene clusters indicated the caspase-1-dependent pyroptosis pathway mediated by the NLRP3 inflammasome to be the core mechanism by which BZYQ intervenes in DN. Pyroptosis, a newly discovered type of programmed cell death closely related to inflammation, is significantly associated with the pathogenesis of DN [25]. The NLRP3 inflammasome consists of NLRP3, apoptosis-associated speck-like protein (ASC), and pro-caspase-1. Upon sensing pathogens or cell damage signals, NLRP3 recruits ASC to form a complex, activating pro-caspase-1 into active caspase-1. Caspase-1 not only induces pyroptosis by cleaving GSDMD, but also promotes the maturation and release of pro-IL-1β and pro-IL-18, thereby driving the inflammatory response [26]. Numerous studies have shown that the expression of pyroptosis-related proteins is significantly increased in renal tissues of DN rat models. Notably, inhibiting the expression of caspase-1 can effectively reduce pyroptosis in renal tubular epithelial cells and podocytes under high-glucose conditions, thereby alleviating renal damage caused by DN [27, 28].

The above studies suggest that inhibiting pyroptosis, which is characterized by inflammatory responses, could be a novel therapeutic strategy and target for DN. In this experiment, after the DN model was established, the expression levels of the inflammasome complex components, including NLRP3, ASC, pro-caspase-1, and caspase-1, were significantly upregulated, and there was an increase in the expression level of pyroptosis substrate GSDMD. Additionally, the expression levels of inflammatory factors IL-18 and IL-1β were markedly elevated, indicating the inflammasome to be activated and pyroptosis to be significantly intensified. After BZYQ treatment, these effects were reversed, showing the activation of the NLRP3 inflammasome to be inhibited, and both the inflammatory response and pyroptosis were effectively controlled.

Inflammation and immune responses interact and influence each other. T-helper (Th) cells, as one of the major immune cell groups, can be categorized into Th1 and Th2 cells based on the cytokines they secrete. Under normal conditions, Th1 and Th2 cells mutually inhibit each other to maintain a relative balance in the immune response. When there is an imbalance between Th1 and Th2 cells, excessive immunoglobulin production can lead to immune-inflammatory diseases. Studies have shown Th1/Th2 imbalance and associated factor changes to be critical mechanisms involved in the pathogenesis of DN. Maintaining Th1/Th2 cell balance can reduce the production of inflammatory cytokines and improve DN [29]. The transcription factors T-bet and GATA3 are key regulators of Th1/Th2 cell balance, playing crucial roles in the development of Th1 and Th2 cells. T-bet promotes Th1 cell development, enhances the expression of IFN-γ, and inhibits Th2 cell differentiation pathways, while GATA3 promotes Th2 cell development, enhances the expression of IL-4, and inhibits Th1 cell differentiation [30, 31]. Our transcriptomic analysis revealed significant differences in T-bet and GATA3 gene expression between the control group and the DN model group. Therefore, regulating T-bet and GATA3 to balance Th1/Th2 cells may be an important pathway for improving DN. Experimental validation confirmed this hypothesis, as T-bet mRNA and protein expression levels were elevated in the model group, indicating a clear shift towards Th1 cells. After BZYQ intervention, T-bet expression significantly decreased, while GATA3 mRNA and protein expression markedly increased, promoting Th2 cell development. This suggests that BZYQ may correct the Th1/Th2 imbalance by regulating the expression of the transcription factors T-bet and GATA3 in DN rats, thereby improving immune dysregulation and reducing inflammation in DN.

Our study has involved several limitations that may affect the generalizability of the results. First, the transcriptomic and bioinformatic analyses showed BZYQ's therapeutic effects on DN to involve multiple pathways. However, our focus was specifically on the inhibition of NLRP3 inflammasome activation and the restoration of Th1/Th2 immune balance, with the aim of thoroughly exploring the mechanisms underlying these pathways. Consequently, we did not independently validate other genes. Future studies can expand the scope of our analysis to provide a more comprehensive understanding of the molecular mechanisms by which BZYQ improves DN, employing approaches, such as proteomics and other omics-based methods. Secondly, further studies are needed to advance the clinical application of BZYQ, including validation in diverse animal models, safety assessments, cost-effectiveness analyses, and preliminary human clinical trials. The therapeutic potential of BZYQ for DN must encourage further collaboration within the scientific community to address these crucial aspects.

## CONCLUSION

In summary, BZYQ has shown a significant therapeutic effect on DN rats. Its mechanism may be primarily related to regulating Th1/Th2 immune imbalance and inhibiting the activation of the NLRP3 inflammasome, thereby suppressing pyroptosis. Furthermore, the transcriptomic and bioinformatic analyses identified several critical pathways and targets, including the extracellular matrix, kidney development, and immune responses, contributing to BZYQ's protective effects on kidney tissues in DN.

This study has not only provided experimental evidence for the mechanism underlying BZYQ’s action, but also highlighted its potential clinical applications in the treatment of DN. These findings may promote the integration of traditional medicine with modern medical practices, offering new hope for DN patients and encouraging further investigation into the clinical and therapeutic potential of BZYQ.

## Data Availability

All data generated or analyzed during this study are included in this published article.

## LIST OF ABBREVIATIONS

DN: Diabetic Nephropathy
ESRD: End-stage Renal Disease
TCM: Traditional Chinese Medicine
BZYQ: Buzhong Yiqi Decoction
DEGs: Differentially Expressed Genes
GO: Gene Ontology
SCr: Serum Creatinine
SD: Sprague-Dawley
UTP: Urinary Protein
FBG: Fasting Blood Glucose
TC: Total Cholesterol
TG: Triglycerides
PBS: Phosphate Buffer Solution
HE: Hematoxylin and Eosin
PPI: Protein-Protein Interaction
KEGG: Kyoto Encyclopedia of Genes and Genomes
GAPDH: Glyceraldehyde-3-phosphate Dehydrogenase
WB: Western Blotting
FBG: Fasting Blood Glucose
