# Supplementary material for: Comprehensive Transcriptomic and Bioinformatic Analysis of the Mechanism of Buzhong Yiqi Decoction in the Improvement of Diabetic Nephropathy
Source: Endocr Metab Immune Disord Drug Targets. 2025 Apr 21;25(13):1099–109. doi: 10.2174/0118715303379062250327184950 (PMC12715389; doi:10.2174/0118715303379062250327184950)
Supplement: Supplementary file 1 [file EMIDDT-25-13-1099_SD1.pdf]

Supplementary Material

Comprehensive Transcriptomic and Bioinformatic Analysis of the Mechanism of Buzhong Yiqi Decoction in the Improvement of Diabetic Nephropathy

Xixu Zhang[1,#], Wei Wei[1,#], Ziyu Liu[1], Hao Gao[2], Fengyi Guo[1], Donglin Liu[2], Yuanping Yin[1,*] and Xiao Yang[1,*]

[1]The Second Affiliated Hospital of Liaoning University of Chinese Medicine, Shenyang, Liaoning 110034, China; [2]Liaoning University of Chinese Medicine, Shenyang, Liaoning 110847, China

Supplementary Table 1. Primer sequence.

Gene	Primer sequence	Bp
NLRP3	Forward primer：CCAGAGCCTCACTGAACTGG	564
	Reverse primer：CCCATGTCTCCAAGGGCATT	
ASC	Forward primer：CACGAGATGCCATCCTGGAC	554
	Reverse primer：TGTCACCAAGTAGGGCTGTG	
Caspase-1	Forward primer：GACCGAGTGGTTCCCTCAAG	108
	Reverse primer：GACGTGTACGAGTGGGTGTT	
GSDMD	Forward primer：GCCCCACTATACCAGACAGC	849
	Reverse primer：CCCGATGGAATGGAGTACGG	
IL-1β	Forward primer：TGTGACTCGTGGGATGATGAC	160
	Reverse primer：CCACTTGTTGGCTTATGTTCTGTC	
IL-18	Forward primer：AGAAGAAGGCTCTTGTGTCAACT	519
	Reverse primer：TCATCCTTCCTTTTCAAAACGAGT	
GATA-3	Forward primer：CTGGAGACGTCTCATTCCCG	596
	Reverse primer：GGGTTGAAGGAGCTGCTCTT	
T-bet	Forward primer：GGGGGAAGAAAAGGAGAAGGG	591
	Reverse primer：CCCCCATCTCTCTCACTTTTTCT	
GAPDH	Forward primer：GAAGGTCGGTGTGAACGGAT	535
	Reverse primer：AGTGATGGCATGGACTGTGG	
